# Impact of a defunctioning ileostomy and time to stoma closure on bowel function after low anterior resection for rectal cancer: a systematic review and meta-analysis

**DOI:** 10.1007/s10151-021-02436-5

**Published:** 2021-04-01

**Authors:** I. Vogel, N. Reeves, P. J. Tanis, W. A. Bemelman, J. Torkington, R. Hompes, J. A. Cornish

**Affiliations:** 1 Department of Surgery, Amsterdam UMC, University of Amsterdam, Amsterdam, The Netherlands; 2 Department of Colorectal Surgery, University Hospital of Wales, Cardiff, UK

**Keywords:** Low anterior resection, Rectal cancer, Ileostomy, Bowel function, Low anterior resection syndrome

## Abstract

**Background:**

Impaired bowel function after low anterior resection (LAR) for rectal cancer is a frequent problem with a major impact on quality of life. The aim of this study was to assess the impact of a defunctioning ileostomy, and time to ileostomy closure on bowel function after LAR for rectal cancer.

**Methods:**

We performed a systematic review based on the preferred reporting items for systematic reviews and meta-analyses (PRISMA) statement. Comprehensive literature searches were conducted using PubMed, Embase and Cochrane databases for articles published from 1989 up to August 2019. Analysis was performed using Review Manager (version 5.3) using a random-effects model.

**Results:**

The search yielded 11 studies (1400 patients) that reported on functional outcome after LAR with at least 1 year follow-up, except for one study. Five scales were used: the Low Anterior Resection Syndrome (LARS) score, the Wexner score, the Memorial Sloan Kettering Cancer Centre Bowel Function Instrument, the Fecal Incontinence Quality of Life scale, and the Hallbook questionnaire. Based on seven studies, major LARS occurred more often in the ileostomy group (OR 2.84, 95% CI, 1.70–4.75, *p* < 0.0001: *I*^2^ = 60%, *X*^2^ = 0.02). Based on six studies, a longer time to stoma closure increased the risk of major LARS with a mean difference in time to closure of 2.39 months (95% CI, 1.28–3.51, *p* < 0.0001: *I*^2^ = 21%, *X*^2^ = 0.28) in the major vs. no LARS group. Other scoring systems could not be pooled, but presence of an ileostomy predicted poorer bowel function except with the Hallbook questionnaire.

**Conclusions:**

The risk of developing major LARS seems higher with a defunctioning ileostomy. A prolonged time to ileostomy closure seems to reinforce the negative effect on bowel function; therefore, early reversal should be an important part of the patient pathway.

**Supplementary Information:**

The online version contains supplementary material available at 10.1007/s10151-021-02436-5.

## Introduction

Low anterior resection (LAR) with total mesorectal excision is the gold standard surgical procedure for patients with resectable primary rectal cancer [1]. When intestinal continuity with a colorectal or coloanal anastomosis is restored, the procedure is often routinely combined with a temporary stoma, to mitigate potential severe sequelae of an anastomotic leak [2]. In general patients have a defunctioning stoma for 3–6 months, however it is not unusual that this period extends beyond 12 months prolonging the negative impact that the presence of an ileostomy has on the patient’s physical, psychological and social wellbeing [3–5].

In patients with a functioning anastomosis, impaired bowel function is a frequent problem with a major impact on quality of life [6, 7]. The symptoms are often referred to as low anterior resection syndrome (LARS), and include; urgency, difficulty emptying and incontinence [8]. A recent meta-analysis found a combined prevalence of major LARS of 41% at least 1 year after stoma closure, when further improvement of the symptoms is unlikely [7]. Radiotherapy and the level of the anastomosis are known to have a negative impact on major LARS, but the presence of an ileostomy and prolonged time to ileostomy closure are mentioned as possible risk factors [9]. However, the literature is inconsistent, with few studies reporting on functional outcome after LAR with and without ileostomy.

Therefore, we performed a systematic review and meta-analysis of the current literature to evaluate the impact of a defunctioning loop ileostomy on bowel function after LAR for rectal cancer. A secondary aim was to assess whether time to stoma closure after the index operation had an effect on bowel function.

## Materials and methods

### Study selection

This systematic review and meta-analysis was conducted in line with the Preferred Reporting Items for Systematic Reviews and Meta-Analysis (PRISMA) guidelines (available at. Prisma-statement.org) [10]. Comprehensive literature searches were conducted using PubMed, Embase and Cochrane databases for articles published from 1989 up to August 2019. The full search strategy is displayed in the Appendix: Table S1–3.

Studies were considered for inclusion if they met the following criteria: (1) patients diagnosed with rectal cancer within 15 cm from the anal verge; (2) patients had LAR with a colorectal or coloanal anastomosis with or without defunctioning ileostomy; (3) assessment of bowel function following LAR with a validated tool; (4) studies were cohort, case matched studies or randomized clinical trials. The exclusion criteria were as follows: (1) reviews, letters, expert opinions, commentaries, case series or case reports; (2) language other than English; (3) lack of the sufficient data or outcomes of interest; (4) duplicate studies; (4) intersphincteric resection.

Two reviewers (IV and NR) independently reviewed titles and abstracts, followed by full text revision. Additionally, the references of relevant studies were hand-searched. Authors of relevant conference abstracts or of studies with missing data were contacted to request more details, 3 authors responded and were able to provide their data sets which made inclusion in our meta-analysis possible [1, 11, 12]. Disagreements were resolved by consensus discussion between the two reviewers (IV and NR).

### Data extraction and quality assessment

Data were extracted independently by two authors (IV and NR) and included the following variables: year of publication, country, study design, number of patients, characteristics of included patients, neoadjuvant treatment, distance of the tumor from the anal verge, proportion of patients undergoing partial mesorectal excision, percentage of anastomotic leaks, number of ileostomies, time to ileostomy closure, length of follow -up and reported scoring systems to assess bowel function.

The primary outcome was bowel function after LAR for rectal cancer in patients with and without an ileostomy and also LARS as a function of time of ileostomy closure.

Risk of bias was assessed using the Newcastle-Ottowa scale for cohort studies and Jadad scoring system for randomized controlled trials [13, 14]. When randomized groups of the randomized controlled trials (RCTs) were not used as described in the RCT, the Newcastle Ottawa quality assessment was used. Two of the authors (IV and NR) performed the quality assessment, and conflicts were discussed to achieve consensus.

### Statistical analysis

Heterogeneity was assessed using the *I*^2^ and *X*^2^ statistics, the data were considered significant if the *p* value (*X*^2^) was < 0.1 with low, moderate, and high for *I*^2^ values of 25%, 50%, and 75%. Analysis was performed using Review Manager (RevMan, version 5.3. Copenhagen: The Nordic Centre, the Cochrane Collaboration, 20) with a random-effects model. The Mantel–Haenzel method was used to calculate the odds ratio and Inverse Variance method was used to calculate the mean difference, both illustrated in forest plots with a 95% confidence interval. When a median time to closure was presented with an interquartile range the our method was used to covert to mean time to closure with a standard deviation.

## Results

**Fig. 1:**
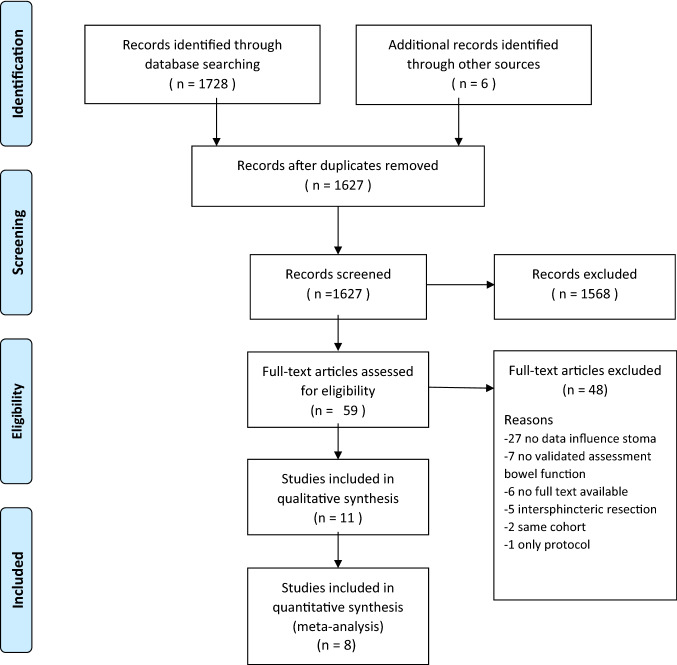
PRISMA flow diagram

In total, 1627 articles were screened on title and abstract (Appendix: Table S1–3). Fifty-nine studies were remained for a full text review. After exclusion of 48 studies, a total of 11 studies with 1400 patients, were included in our systematic review (Fig. 1). Seven studies were cohort studies and four studies were secondary analysis of randomized controlled trials. One randomized controlled trial was used as a cohort study [15]. The assessment for methodological quality and risk of bias is listed in the Appendix: Table S4.

### Patient characteristics

Baseline characteristics of the studies are listed in Table 1. All patients had LAR for rectal cancer with a colorectal or coloanal anastomosis. Ten studies mentioned the proportion of patients receiving neoadjuvant radiotherapy, which ranged from 26% to 97% [1, 11, 12, 15–21]. The majority were mid and proximal rectal cancers (5–15 cm from the anal verge), with varying proportions of distal tumors (< 5 cm) ranging from 0 to 37% (Table 1). Five studies reported the number of patients that underwent a total or partial mesorectal excision, 3 studies reported 100% total mesorectal excision (TME), partial mesorectal excision was mentioned in 2 studies, and constituted 27% and 50% of their study population, respectively [17, 19]. In total, 61.2% of patients (*N* = 857, 29% to 100% in the included studies). received an ileostomy, In one study, by Keane et al., 100% of the patients received an ileostomy [11]. Eight studies reported on acute anastomotic leaks with an incidence of 0–12.7% [1, 11, 12, 15, 17, 19–21].

**Table 1:** Baseline characteristics

Study	Country	Design	Number of patients	Median age (range)	*N* female (%)	Tumor height, distance from anal verge *N* (%), cm		Number of TME (%)	Neo-adjuvant treatment (%)	N stoma (%)	Anastomotic leak (%)	Reported scores
Keane [11]	Australia	Secondary analysis RCT	82	67 (53–73)	34 (41.5)	5–9 10–15 ≥ 15	42 (51%) 39 (48%) 1 (1%)	82 (100)	21 (25.6)	82 (100)	0	LARS, MSKCC BFI
Sun [15]	China	Secondary analysis RCT	220	57 (21–77)	75 (34.0)	< 5 5–10 > 10	81 (37%) 128 (58%) 11 (5%)	220 (100)	214 (97.3)	170 (77.3)	28 (12.7)	LARS
van Heinsbergen [16]	Netherlands	Retrospective	163	72 (47–86)	64 (39.3)	< 5 5–9.9 10–14.9	12 (8%) 82 (50%) 69 (42%)	–	135 (82.8)	128(78.5)	–	LARS
Jiminez-Gomez [17]	Spain	Cross sectional	184	–	61 (33.2)	–		134 (72.8)	122 (66.3)	97 (52.7)	16 (8.7)	LARS
Gadan [12]	Sweden	Secondary analysis RCT	87	64 (32–64)	38 (44)	Median 5 (range 2.5–7)		–	75 (86.2)	47 (54.0)	11 (12.6)	LARS
Hughes [1]	UK	Cross sectional	68	67 (30–88)	19 (27.9)	< 8 > 8	22 (32%) 34 (50%)	–	19 (27.9)	37 (54.4)	5 (7.0)	LARS
Jiminez-Rodriguez [18]	Spain	Cross sectional	150	–	57 (38.0)	Median 13 (range 2–15)		–	93 (62.0)	43 (28.6)	–	LARS
Sturiale [22]	Italy	Cross sectional	60	66 (29–83)		< 5 5–10 10–12	28 (30%) 39 (42%) 26 (28%)	–		21(35)	–	LARS
Bondeven [19]	Denmark	Prospective	125	64 (39–84)	46 (36.8)	0–5 > 5–10 > 10–15	7 (5%) 47 (38%) 71 (57%)	63 (50)	25 (20.0)	84(67.2)	0	LARS
Walma [20]	Netherlands	Retrospective	80	63 (57–69)	27 (33.7)	≤ 5 5– 10 > 10–15	9 (11%) 42 (43%) 29 (37%)	125 (100)	62 (77.5)	58 (72.5	5 (6.3)	Wexner, FIQoL
Lindgren [21]	Sweden	Secondary analysis RCT	181	67 (33–85)	86(47.5)	Median 5 (range 2–7)		–	146 (80.7)	90(49.7)	20 (11.0)	Hallbook

*RCT* randomized controlled trial; *TME* total mesorectal excision; *LARS* low anterior resection syndrome; *MSKCC–BFI* Memorial Sloan Kettering Cancer Centre Bowel Function Instrument; *FIQoL* Fecal Incontinence Quality of Life scale; *Hallbook* bowel function questionnaire by Hallbook

### Functional outcomes

Five different scales were used to report on bowel function: the LARS score, Wexner score, bowel function questionnaire by Hallbook, Memorial Sloan Kettering Cancer Centre Bowel Function Instrument (MSKCC-BFI) and Fecal Incontinence Quality of Life (FIQoL) scale. Nine studies used the LARS score and the remaining scores were used by only 1 study each, specified in Table 1. An explanation of the different scoring systems is in the Appendix: Table S5 [23–27]. Median time to stoma closure was reported in 8 studies and varied from under 3.4 to 19 months (Table 3) [1, 11, 12, 15–18, 21]. Six studies reported the number of patients that could not be included in the assessment of bowel function because of a permanent stoma, ranging from 6.3% to 27.7% of the total number of patients that were assessed for eligibility [11, 12, 18, 20–22]. The number of patients not eligible for inclusion because of death was reported in five studies and ranged from 5.9% to 49.6% [11, 12, 18, 20, 22] .

### LARS score

Nine studies reported on LARS score in 1139 patients with a median follow-up time of 54.3 months (range 8.2–164.4 months) [1, 11, 12, 15–19, 22]. With the exception of Hughes et al. [1], follow-up time was more than 1 year in all studies. Overall 48.3% of the patients reported major LARS (*n* = 550), while minor LARS or no LARS were reported in 22.0% and 29.7% of the patients respectively (Table 2). In six studies, major LARS was significantly more frequent in the group of patients that received an ileostomy in univariate analysis [12, 15–19]. An ileostomy was also associated with increased risk of major LARS in multivariate analysis after correction for neoadjuvant therapy and tumor height as confounding factors in two studies [15, 16]. Higher rates of major LARS in defunctioned patients were also found in the other studies, but the results were not statistically significant [1, 22]. In the study by Keane et al., all patients were defunctioned, which made the assessment of the impact of a stoma impossible.

**Table 2:** LARS score after LAR for rectal cancer

Author	No LARS (%)	Minor LARS (%)	Major LARS (%)	Time from surgery to LARS median months (range)
Keane [11]	16 (20)	12 (15)	54 (65.9)	49 (24–77)*
Sun [15]	27 (12.3)	74 (33.6)	119 (54.1)	40.2 (23–87)
van Heinsbergen [16]	47 (28.8)	29 (47.3)	87 (53.4)	62.4 (28–100)
Jiminez-Gomez [17]	44 (23.9)	36 (19.6)	104 (56.2)	45.7 (31–64)*
Gadan [12]	24 (27.5)	17 (19.5)	46 (52.9)	141.8 (117–177)
Hughes [1]	18 (26)	12 (18)	38 (58)	8.2 (0.6–55)
Jimiez-Rodriquez [18]	82 (54.7)	26 (17.3	42 (28.0)	36 (12–60)
Sturiale [22]	32 (53.3)	15 (25)	13 (21.7)	164.4 (131–216)
Bondeven [19]	48 (38)	30 (24)	47 (38)	18 (12–24)
Total	338 (29.7)	251 (22.0)	550 (48.3)	

*LARS*  low anterior resection syndrome

^*^Presented with interquartile range instead of range

**Fig. 2:**
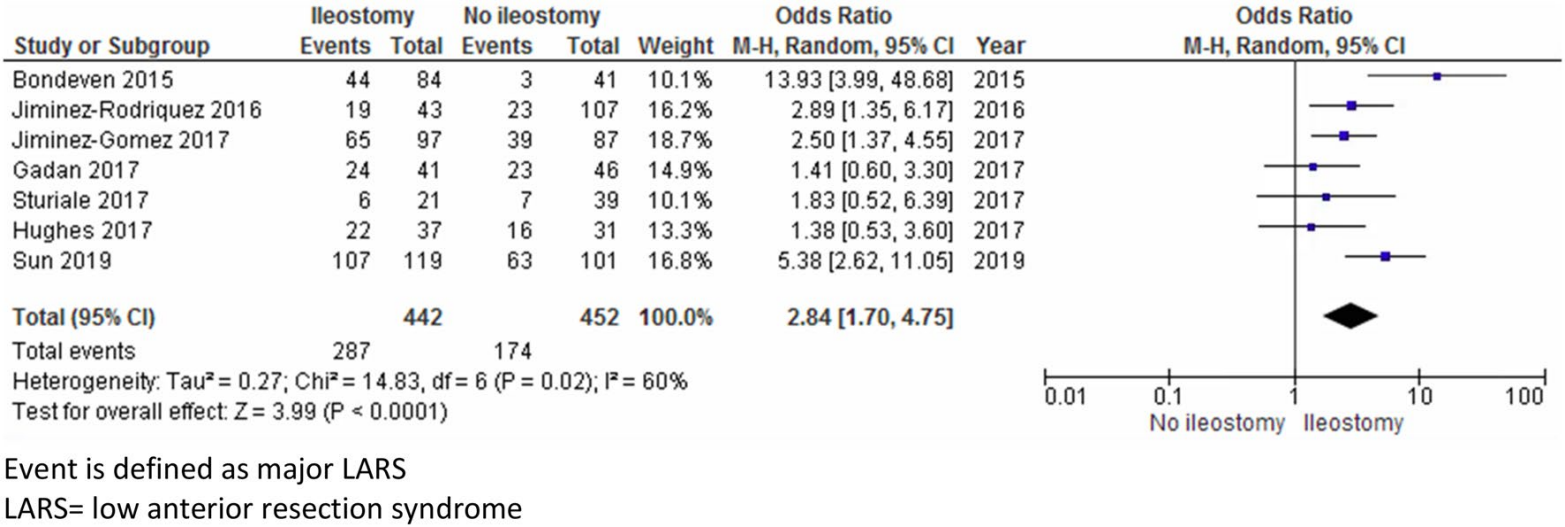
Prevalence of major LARS in ileostomy and no ileostomy group. Event is defined as major LARS. *LARS* low anterior resection syndrome

A total of 894 patients from seven studies were included for the pooled analysis after excluding van Heinsbergen et al. (no separate data on presence of stoma available for rectal cancer population); major LARS occurred significantly more often in the ileostomy group compared to the non defunctioned patients with an OR of 2.84 and moderate levels of heterogeneity between the included studies (95% CI, 1.70–4.75 *p* < 0.0001: *I*^2^ = 60%, *X*^2^ = 0.02), Fig. 2 [1, 12, 15, 17–19, 22].

### Impact of timing of stoma closure

Six studies evaluated the influence of timing of ileostomy closure on LARS score (Table 3) [1, 11, 12, 15, 17, 18]. Hughes et al. showed in multivariate analysis that ileostomy reversal within 6 months after initial surgery was protective against major LARS (OR 0.2, 95% CI, 0.1–0.3, *p* < 0.01) and that reversal after 1 year was associated with increased risk of major LARS (OR 3.7, CI 95%, 1.1–13.1, *p* = 0.03) [1]. An increased risk of major LARS after longer time to ileostomy closure was also found in 4 other studies, but without a statistically significant difference [11, 12, 15, 17]. Only one study reported no influence of timing of stoma closure on LARS score; in this trial there was only a 2 week difference in the time to closure in the major LARS vs no LARS group and the mean time was 15.1 months vs 15.6 months respectively [18].

**Table 3:** Reported time to ileostomy closure and functional score

Study	Reported scores	Dropout due to permanent stoma (%)*	Dropout due to death (%)*	*N* patients with stoma (%)	Median months to stoma closure (range)	Follow-up time months (range)
Keane [11]	LARS, MSKCC BFI	7 (6.3)	12 (10.7)	82 (100)	3.4 (0.3–12.2)	49 (24–77)**
Sun [15]	LARS	–	–	170 (77.3)	5.3 (0.6–22.9)	40.2 (23–87)
van Heinsbergen [16]	LARS	–	–	128 (78.5)	4.5 (0.2–16.3)	62.4 (28–100)
Jiminez-Gomez [17]	LARS	–	–	97 (52.7)		45.7 (31–64)**
Gadan [12]	LARS	27 (11.5)	109 (27.7)	47 (54.0)	5.1 (0.4–19.6)	141.8 (117–177)
Hughes [1]	LARS			37 (54.4)	7.0 (1.6–11.9)	8.2 (0.6–55)
Jiminez-Rodriguez [18]	LARS	109 (27.7)	105 (26.6)	43 (28.6)	15 (3–31)	36 (12–60)
Sturiale [22]	LARS	39 (10.3)	177 (46.6)	21 (35)	–	164.4 (131–216)
Bondeven [19]	LARS	–	–	84 (67.2)	–	18 (12–24)
Walma [20]	Wexner, FIQoL	21 (8.7)	14 (5.9)	58 (72.5	19 (10–29)	19 (10–29)**
Lindgren [21]	Hallbook	33 (24.8)	–	90 (49.7)	6 (1–21)	12 (7–24)

*LARS* low anterior resection syndrome; *MSKCC–BFI* Memorial Sloan Kettering Cancer Centre Bowel Function Instrument; *FIQoL* Fecal Incontinence Quality of Life scale; *Hallbook* bowel function questionnaire by Hallbook

^*^Percentage of total number of patient in cohort that received an anterior resection for rectal cancer and were assessed for eligibility bowel function questionnaire

^**^Median presented with interquartile range instead of range

**Fig. 3:**
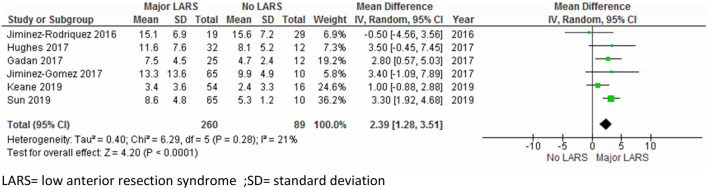
Time to stoma closure major LARS vs no LARS. *LARS* low anterior resection syndrome; *SD *standard deviation

Six studies, on 719 patients, were included in pooled analysis on the influence of timing of ileostomy closure on prevalence of major LARS. Mean time to ileostomy closure ranged from 2.4 to 15.6 months with a mean difference of 2.39 months (95% CI, 1.28–3.51, *p* < 0.0001: *I*^2^ = 21%, *X*^2^ = 0.28) in the major vs. no LARS group with low levels of heterogeneity between the individual studies [1, 11, 12, 15, 17, 18] (Fig. 3). Patients with major LARS had an average time of ileostomy closure 10 weeks later than those with no LARS.

### Wexner and FIQoL Scale

Walma et al. was the only study that reported on Wexner score and FIQoL. They reported that an ileostomy was an independent predictor for impaired FIQoL (OR −0.524, 95% CI, −1.072 to −0.021, *p* = 0.041) and in the univariate analysis the Wexner score was negatively influenced by an ileostomy. Ileostomy reversal within 3 months after the index operation showed significantly better FIQoL (median 15, IQR 13.1–16 vs. 12, IQR 10.5–13.9; *p* = 0.001) and Wexner score (median of 5, IQR 2.5–8 vs. 10.5, IQR 6–13.8; *p* < 0.001) than reversal after 3 months [20].

### MSKCC-BFI

In the secondary analysis of the EASY trial, both the LARS score and the MSKCC-BFI score were used to assess bowel function in patients with early stoma closure within 2 weeks and after 16 weeks. Patients in the early closure group showed higher BFI scores compared to late closure group, which indicates better bowel control, although this was not statistically significant (median BFI score 71, IQR 59–75 vs. 63 IQR 60–70 *p* = 0.27) [11]. Participants in the late closure group did have a significantly worse BFI score on the urgency/soiling subscale (median score early group 14 vs late group 17, *p* = 0.017). A strong negative correlation was noted between the two scoring systems (ρ = −0.72, *p* < 0.001).

### Bowel function questionnaire by Hallbook

Lindgren et al. used the bowel function questionnaire by Hallbook to assess anorectal function after LAR [21]. The presence of an ileostomy did not affect anorectal function, evaluated 1 year after stoma closure. However, no overall scores were presented, only scores of the different subscales. Median scores were the same in each group regarding need for medication, evacuation difficulties, fragmentation of bowel movements, incontinence and effects on wellbeing. On the urgency subscale patients with a stoma had a higher incidence of urgency compared to patients without a stoma, 35.5% vs. 25.4%, although not statistically significant.

## Discussion

In this systematic review and meta-analysis, we evaluated the impact of a defunctioning stoma, and time to stoma closure on bowel function after LAR for rectal cancer. The risk of developing major LARS was higher when a patient has had a defunctioning ileostomy compared to patients without an ileostomy. A prolonged time to ileostomy closure was associated with an increased incidence of major LARS.

The included studies were from Australia, China, the Netherlands, the United Kingdom, Spain and Sweden and therefore represent a wide geographical area. All included studies, with the exception of the study by Hughes et al. [1], have at least a follow-up time of 1 year. It has been demonstrated that bowel dysfunction improves over time, but the improvement is limited after one year, thus these results reliably represent bowel function outcome after LAR [21, 28].

A recent systematic review compared bowel function between patients undergoing LAR with and without ileostomy and outcomes for early versus later ileostomy closure [29]. Keane et al. included only four studies that assessed bowel function, and three of the four studies were from the same cohort of patients [11, 12, 18, 30]. A pooled analysis was performed with two of the included studies which showed a twofold increase in the rate of major LARS in patients with an ileostomy (OR 1.96, 95% CI, 1.1–3.5, *p* = 0.02). This is in line with the results of our analysis, although through collection of raw data we were able to perform a pooled analysis that included six studies and showed an even stronger effect of an ileostomy on the risk of major LARS (OR 2.84, 95% CI, 1.70–4.75, *p* < 0.0001). Keane et al. report that they were unable to perform a meta-analysis on the influence of timing of ileostomy closure on bowel function.

The association between LARS and presence of an ileostomy could be due to a variety of reasons; an ileostomy is more likely to be created in low anastomoses and those patients are more likely to receive neoadjuvant therapy, which have both been shown to be associated with LARS [9]. The contribution of the length of the remnant rectum to post-operative function might be due to the interruption of the complex physiology of bowel motility and rectoanal coordination [19]. Suggested mechanisms for how radiotherapy impacts bowel function include nerve damage, impairment of the anal sphincter, and decreased neorectal compliance caused by radiation-induced fibrosis [15]. Nonetheless an ileostomy might have an independent influence on the LARS score. In the EASY trial (2019), the risk of major LARS was increased when there was a prolonged time to closure and results remained similar after adjusting for tumor height and use of radiotherapy [11] and several studies reported that both radiotherapy, tumor height as well as the presence of a defunctioning stoma were independent predictors of worse LARS score [15, 16].

Anastomotic leak has been mentioned as a risk factor for developing major LARS [4]. Patients who have an anastomotic leak will often have an ileostomy for a more prolonged period before closure. Of the included studies in our review, 5 assessed the association between an anastomotic leak and LARS where none of the studies confirmed a correlation [1, 12, 15, 17, 22]. In addition the prevalence of major LARS in almost 50% of the patients is much higher than the reported percentages of anastomotic leak, ranging from 0%–12.7% in the included studies. The low numbers of anastomotic leak might contribute to the lack of a significant impact on LARS score due to a type II error. Furthermore, anastomotic leak rates might be underreported in the presence of a covering stoma, when an asymptomatic leak might be present. Results from the Dutch SNAPSHOT study showed an increase in anastomotic leak within 30 days from 8.2% to 13.4%, when retrospectively checking data from the registry, which increased to 20% at end of follow-up after occult leaks were found during stoma reversal [31].

Other findings pertaining to LARS include changes in the functional state of the pelvic floor and the sphincter complex during and after a LAR [32]. When not used for a prolonged period of time, in a defunctioned state, pelvic floor muscles are likely to lose strength and bowel function might be impaired as a result. This is in line with a systematic review by Visser et al. that demonstrated that the use of pelvic floor rehabilitation was useful for improving functional outcome after LAR [33].

Another reason for the association between an ileostomy and major LARS may be due to alterations in colonic environment and microbiota. There is loop ileostomy-mediated fecal stream diversion which alters microbiota composition, which in turn affects intestinal epithelial cell turnover and consequently impacts on intestinal structure and function [34]. One study comparing colonic flora of patient with and without an ileostomy found that the abundance of several types of bacteria differed significantly between the ileostomy and control groups [35]. This could be an explanation for the presence and duration of an ileostomy being an influence on the prevalence of major LARS.

Most surgeons aim to reverse a stoma within 2–4 months after initial surgery, but the scheduling of stoma closure is extremely variable among hospitals [11, 36]. There are reports that it is safe to close a defunctioning stoma within 2 weeks after surgery, with a similar rate in post-operative complications and anastomotic leaks compared to delayed closure [37]. In the United Kingdom, the national bowel cancer audit reported that up to 40% of patients had a delay in closure of more than 18 months, which is confirmed in the CLOSE-IT trial [5]. None of the included studies reported on reason for delay in stoma closure. In previous reports, adjuvant chemotherapy is mentioned as a potential reason for delay, but when comparing patients who underwent closure of an ileostomy either during adjuvant chemotherapy or after chemotherapy a large Korean study found that post-operative hospital stay, post-operative complications, and disease free survival were equal in both groups [38]. While clinical factors might preclude timely closure, different healthcare systems with different reimbursement/funding and logistics will have a varying average in their time to stoma closure [39]. Currently there is a lack of guidance on the optimal timing of stoma closure.

There are some limitations to this systematic review. None of the included studies had a pre-study power calculation performed for this review’s primary outcome variable. Within the randomized controlled studies, the assessment of bowel function after an ileostomy was part of a post-hoc analysis. The majority of the studies included were cohort studies without clear guidelines on when to defunction the anastomosis with a risk of selection bias. The decision to create an ileostomy is often left to the surgeon's discretion, and patients with an ileostomy might have a lower anastomosis, more complex surgery, and/or neoadjuvant therapy. This could explain the moderate heterogeneity between the included studies in our meta-analysis comparing LARS in patients with and without ileostomy. Furthermore, none of the studies reported on the effect of patient related factors such as body mass index and age on bowel function, nor was the influence of operative time addressed as indicator of the complexity of the surgery.

Finally there were five different tools used to assess bowel function though many more are described in the literature, which makes comparison between different studies difficult. The most commonly reported was the LARS score, a score specifically designed for rectal cancer patients after LAR [40]. However this score is thought to underestimate evacuatory dysfunction and may not accurately assess the impact of symptoms on individual patient’s quality of life [41]. The interpretation of the LARS score results is also limited by the findings that major LARS is also common in the general population without rectal cancer, especially in women between 50 to 79 years old [42]. A new LARS score is being developed but so far optimal scoring systems to assess bowel function after LAR are lacking [41].

The cause of poor bowel function after rectal cancer surgery is multifactorial. Further studies assessing the pathophysiology of the association an ileostomy and major LARS and influence of timing on ileostomy closure are required with bowel function as primary outcome of the study.

## Conclusions

Based on this meta-analysis, the risk of having major LARS seems higher with a defunctioning ileostomy, and prolonged time to ileostomy closure seem to have a negative effect on bowel function. Pros and cons of routine diversion during LAR should be reconsidered.

## Supplementary Information

Below is the link to the electronic supplementary material.

Supplementary file1 (DOCX 34 KB)
