# Supplementary material for: Impact of a defunctioning ileostomy and time to stoma closure on bowel function after low anterior resection for rectal cancer: a systematic review and meta-analysis
Source: Tech Coloproctol. 2021 Apr 1;25(7):751–60. doi: 10.1007/s10151-021-02436-5 (PMC8187190; doi:10.1007/s10151-021-02436-5)
Supplement: Supplementary file 1 — Supplementary file1 (DOCX 34 KB) [file 10151_2021_2436_MOESM1_ESM.docx]

**Appendix**

Date of search: 13-08-2019

**Supplementary Table 1**

Database(s): **Medline (via OVID)** 1990-present

| **#** | **Searches** |
| --- | --- |
| 1 | (Rectal cancer OR rectal neopl* OR colorectal cancer OR colorectal neopl* OR colorectal carcinoma OR rectal carcinoma OR rectal tumor OR colorectal tumor OR rectal tumour OR colorectal tumour). ti,ab,kw. |
| 2 | (ileostomy OR surgical stoma OR diverting stoma) ti,ab,kw. |
| 3 | (Anterior resection OR total mesorectal excision) ti,ab,kw. |
|  | 1 and 2 and 3 |
| **Total hits** | **841** |

**Supplementary Table 2**

Database(s): **Embase Classic+Embase (via OVID)** 1990-present

| **#** | **Searches** |
| --- | --- |
| 1 | (Rectal cancer OR rectal neopl* OR colorectal cancer OR colorectal neopl* OR colorectal carcinoma OR rectal carcinoma OR rectal tumor OR colorectal tumor OR rectal tumour OR colorectal tumour). ti,ab,kw. |
| 2 | (ileostomy OR surgical stoma OR diverting stoma) ti,ab,kw. |
| 3 | (Anterior resection OR total mesorectal excision) ti,ab,kw. |
|  | 1 and 2 and 3 |
| **Total hits** | **863** |

**Supplementary Table 3**

Database(s): **Cochrane Library** 1990-present

| **#** | **Searches** |
| --- | --- |
| 1 | Mesh descriptor: [Rectal neoplasm] explode all trees |
| 2 | (Rectal cancer OR rectal neopl* OR colorectal cancer OR colorectal neopl* OR colorectal carcinoma OR rectal carcinoma OR rectal tumor OR colorectal tumor OR rectal tumour OR colorectal tumour). ti,ab,kw. (word variations have been searched) |
| 3 | 1 OR 2 |
| 4 | Mesh descriptor: [Ileostomy] explode all trees |
| 5 | (ileostomy OR surgical stoma OR diverting stoma) ti,ab,kw. (word variations have been searched) |
| 6 | 4 OR 5 |
| 7 | (Anterior resection OR total mesorectal excision) ti,ab,kw. (word variations have been searched |
| 8 | 3 and 6 and 7 |
| **Total hits** | 24 (2 Cochrane reviews, 22 trials) |

**Supplementary table 4 Methodological quality and risk of bias**

| **Study** | **Jadad**  **Score** | **New** | **Ottawa** | **Assessment** | **Bias** |
| --- | --- | --- | --- | --- | --- |
|  |  | **Selection (0-4)** | **Comparability (0-2)** | **Outcome (0-3)** | **Total (0-9)** |
| Keane 2019^(11)^ | 6 |  |  |  |  |
| Sun 2019^(15)^ |  | *** | ** | ** | 7 |
| van Heinsbergen 2018^(16)^ |  | ** | * | ** | 5 |
| Jiminez-Gomez 2017^(17)^ |  | *** | * | ** | 6 |
| Gadan 2017^(12)^ | 6 |  |  |  |  |
| Hughes 2017^(1)^ |  | *** | * | * | 5 |
| Jiminez-Rodriguez 2016^(18)^ |  | *** | * | ** | 6 |
| Sturiale 2016^(22)^ |  | *** | * | * | 5 |
| Bondeven 2015^(19)^ |  | *** | * | * | 5 |
| Walma 2015^(20)^ |  | **** | * | * | 6 |
| Lindgren 2011^(21)^ | 6 |  |  |  |  |

**Supplementary table 5 Explanation scoring systems**

| **Scoring system** | **Questions** | **Interpretation scores** |
| --- | --- | --- |
| LARS score^(38)^ | Five questions, each with associated response categories based on the frequency of symptom occurrence or number of bowel motions. | Each response is weighted based on the impact on quality of life. A LARS score of 0–20 represents no LARS, 21–29 minor LARS, and 30–42 major LARS |
| MSKCC- BFI^(39)^ | 18 questions that are divided into subscales: dietary, soiling/urgency and frequency. | The BFI gives an overall score between 0 and 100, with a higher score representing better function |
| Wexner score^(40)^ | 5 question with associated response catagories regarding different anal incontinence presentations: Gas/Liquid/Solid/Pad use/Need for lifestyle alterations | A higher Wexner score indicates more symptoms of fecal incontinence on a scale from 0 to 20. |
| FIQoL^(41)^ | 29 questions with 4 subscales to rate quality of life across lifestyle, coping/behavior, depression/self-perception and embarrassment catagories | Subscale scores range from 1 to 5 and are the average response to all items on the scale. A lower score indicates a lower functional status and related quality of life |
| Hallbook^(42)^ | 10 questions assessing various aspects of anorectal function, including stool frequency, medication, evacuation difficulties, fragmentation of bowel movements, incontinence, urgency, effect on well-being, and whether the patient would prefer a permanent stoma if it helped with bowel problems. | Questions are forming a scale from 3 points (best continence) to 12 points (worst continence) |

LARS, low anterior resection syndrome; MSKCC-BFI , Memorial Sloan Kettering Cancer Centre Bowel Function Instrument; FIQoL, Fecal Incontinence Quality of Life scale;

Hallbook, bowel function questionnaire by Hallbook
